# In the Eye of the Cytokine Storm: A Tale of SARS-CoV-2-Induced Acute Macular Neuroretinopathy

**DOI:** 10.7759/cureus.36797

**Published:** 2023-03-28

**Authors:** Sujana Reddy, Alizah Garvin, Jeffrey L Weeks, Matthew R West

**Affiliations:** 1 Internal Medicine, East Alabama Health, Auburn, USA; 2 Internal Medicine, Edward Via College of Osteopathic Medicine (VCOM), Auburn, USA; 3 Ophthalmology, Weeks Cataract and Eye Center, Opelika, USA; 4 Ophthalmology, Retina Specialists of Alabama, LLC, Birmingham, USA

**Keywords:** cytokine storm, sars-cov-2, covid-19, optical coherence tomography, oct (optical coherence tomography), covid-19 pandemic, microvascular, cytokine storm syndrome, acute macular neuroretinopathy

## Abstract

Acute macular neuroretinopathy (AMN) commonly affects young to middle-aged females and is considered a relatively rare retinal disease, and the etiology is complex. Advances in multimodal imaging provide a better characterization of retinal disorders and have helped identify that one of the etiologies of AMN is microvascular in nature. This case is clinically relevant as it adds to the literature that the pathophysiology of AMN is vascular-driven. Our case is a 24-year-old Black female with no past medical history, the only medication she was taking was an oral contraceptive pill, who presented to the emergency room with a 24-hour history of left central field vision loss and endorsed a recent upper respiratory tract infection preceding the acute vision loss. It was subsequently found on admission that the patient tested positive for and had a SARS-CoV-2 infection. A retina specialist performed optical coherence tomography (OCT), which showed disruption in the outer segment junction, including the ellipsoid zone and outer plexiform. The use of multimodal imaging like OCT helped confirm AMN; therefore, prompt examination by ophthalmology is critical to confirm a correct diagnosis. This patient’s vision improved and remained stable five months later. This case demonstrates that, like other viruses, SARS-CoV-2 has the potential to cause retinal disease complications such as AMN. These findings reinforce and add to the current literature that SARS-CoV-2 can cause multiple-organ system dysfunction at a vascular level through immune-mediated pathways.

## Introduction

Acute macular neuroretinopathy (AMN) is a rare macular disorder that was discovered in the Netherlands in 1975 by Dr. Bos and Dr. Deutman. These physicians noted this neuroepitheliopathy affecting the retinal layers of the macula in four women, aged 24 to 35 years, who were all on oral contraceptives [1]. AMN can result in both temporary and permanent vision loss. Fundus findings of AMN include one or more acute petaloid-shaped scotomas that are often described as reddish-brown, wedge-shaped lesions located around the fovea of the retina. These “flower petal” perifoveal lesions usually become visible shortly after the onset of symptoms but can be visible days to months later, and in some cases can persist indefinitely [2]. A comprehensive review of AMN completed in 2016 included 101 patients from 1975 to 2014; this study concluded that AMN presents primarily in young females with over half of those cases occurring between the ages of 21 and 30 years [3]. Some risk factors associated with AMN include infections such as influenza or severe acute respiratory syndrome coronavirus 2 (SARS-CoV-2), oral contraceptive pill use, vaccinations, intravenous epinephrine or ephedrine, intravenous contrast, systemic shock, trauma such as head injury, caffeine, and pro-thrombin-associated antiphospholipid antibodies [2-4].

The primary AMN symptoms are paracentral scotomas, or blind spots, in an otherwise normal visual field. Other symptoms that can arise include acute loss of vision, blurry vision, floaters, and metamorphopsia [2,4]. The exact cause of AMN is yet to be determined; however, recent research has found that a vascular defect could be contributing to the symptoms. A recent case in 2019 showed that AMN could have microvascular involvement of both the superficial and deep capillary plexus of the retina [5]. Other articles also postulate that microthrombi leading to ischemia of the retina can contribute to the pathophysiology of AMN [6].

Recent literature has illustrated a rise in AMN since the start of the coronavirus disease 2019 (COVID-19) pandemic in 2020, caused by SARS-CoV-2. A disease that was known to be rare, where providers saw roughly less than one case of AMN per year, suddenly was seen more often; providers and researchers began observing a relationship between increased AMN incidence and SARS-CoV-2 infection and vaccination. A case series completed in 2022 included 14 cases of patients diagnosed with AMN following recent SARS-CoV-2 infection or vaccination, further supporting the theory of the possible inflammatory etiology of the virus or vaccines triggering a thromboembolic event or vascular cause, leading to ischemia to the deep retinal capillary plexus [7]. This case is specific to a 24-year-old female with no past medical history, other than a history of oral contraceptive pills, who presented with central vision loss of the left eye three days after an upper respiratory infection, caused by SARS-CoV-2. This case adds to the current literature by illustrating how a combination of two risk factors of SARS-CoV-2 infection and contraceptive medication led to AMN in an otherwise young, healthy female. This case provides additional support that these key risk factors are associated with an increase in the number of cases of AMN during the SARS-COV-2 pandemic.

## Case presentation

A 24-year-old Black female with no significant past medical history arrived at the emergency room with a two-day history of left monocular painful vision loss. She reported running fevers of unknown temperature, malaise, and sore throat three days prior with a two-day history of a clear productive cough. The day before coming to the ER, she awoke from a nap and noted a frontal headache (no prior history of migraines) and left central vision loss (darkened vision in the center) along with retrobulbar pain, which progressed later that evening to decreased vision in both eyes, with left eye worse than right. That night she took over-the-counter Tylenol and Zicam without any relief of symptoms. The following day, her vision did not improve, and she still had pain with extraocular movements, so she went to the urgent care center that directed her to the ER for concern of potential optic neuritis. Her only home medication was an oral contraceptive pill, ethinyl estradiol-norethindrone (Tarina Fe 1/20), once a day for the last six years. She endorsed diarrhea (three to four bowel movements a day) that began on the day of admission and lasted roughly four days. Imodium was given as needed during her hospital stay. She denied redness, tearing, or discharge of the left eye. She denied any sensory deficits, previous vision loss, or a family history of multiple sclerosis. Visual acuity on admission in the left eye (OS) was 20/70 and 20/20 in the right eye (OD).

A battery of tests was obtained on admission, and relevant findings included a comprehensive metabolic panel (CMP) (mild hypokalemia at 3.5 mmol/L), complete blood count (CBC) with differential (leukopenia at 3.3 x 103/μL with a low absolute neutrophil count of 1800 per microliter and with elevated monocytes at 13.5%), D-dimer within normal limits at <150 ng/dL, and a C-reactive protein (CRP) (elevated at 2.5 mg/dL, which trended down to 0.6 mg/dL during hospital stay). Respiratory multiplex “BioFire” polymerase chain reaction (PCR) was positive for the SARS-CoV-2 virus without other concomitant infections (Table 1). The patient was vaccinated with two doses of the Pfizer mRNA vaccine about one year and three months prior. CT angiogram of the head with contrast showed no acute findings, patent intracranial vasculatures, and no evidence of acute intracranial thrombosis or occlusion. Neurology was consulted and ordered an MRI of the brain, face, neck, and orbits with and without contrast, which showed no acute findings or T2/fluid-attenuated inversion recovery (FLAIR) signal abnormality of the optic nerves, optic chiasm, or optic tracts. Given the clinical course, the patient was initially diagnosed with presumed optic neuritis secondary to SARS-CoV-2 infection. The patient declined lumbar puncture. She was started on 1 gram of methylprednisolone once a day for three days. On the second day of IV steroid treatment, the patient noted improvement in left eye pain but no major improvement in vision. Infectious disease was also consulted and the patient was given a two-day course of intravenous infusion of remdesivir 200 mg on day one and 100 mg on day two.

**Table 1:** Rapid BioFire RP2.1 respiratory PCR for 22 pathogens confirming positive results for SARS-CoV-2. PCR: polymerase chain reaction.

Resp. panel	
COVID-19 (SARS-CoV-2)	Detected
Adenovirus	Not detected
Coronavirus HKU1	Not detected
Coronavirus NL63	Not detected
Coronavirus 229E	Not detected
Coronavirus OC43	Not detected
Human metapneumovirus	Not detected
Human rhinovirus/enterovirus	Not detected
Influenza A	Not detected
Influenza B	Not detected
Parainfluenza 1	Not detected
Parainfluenza 2	Not detected
Parainfluenza 3	Not detected
Respiratory syncytial virus	Not detected
Bordetella pertussis	Not detected
Bordetella parapertussis	Not detected
Chlamydia (Chlamydophila) pneumoniae	Not detected
Mycoplasma pneumoniae	Not detected

Ophthalmology was consulted and noted a slight color vision deficit with the Ishihara color test (11/11 OD, 9/11 OS). Intraocular pressure (14 OD, 18 OS) was within the normal range of 10 to 21 millimeters of mercury (mmHg). On a fundoscopic exam, there was no optic disc edema or relative afferent pupillary defect (RAPD). Macula was flat and sharp with good reflex OD. There was, however, a petaloid appearance in the left macula with mild reflex suggesting a macular process. Extraocular muscles were intact in both eyes (OU), and admission pain with lateral gaze deviation of her left eye improved (no diplopia with any directional gaze of either eye), pupils were equally round and reactive to light and accommodation, and the visual acuity exam with Snellen chart showed 20/20 vision in the right eye (OD) and 20/50 in the left eye (OS). Vision remained stable during the four-day hospital course.

**Figure 1:**
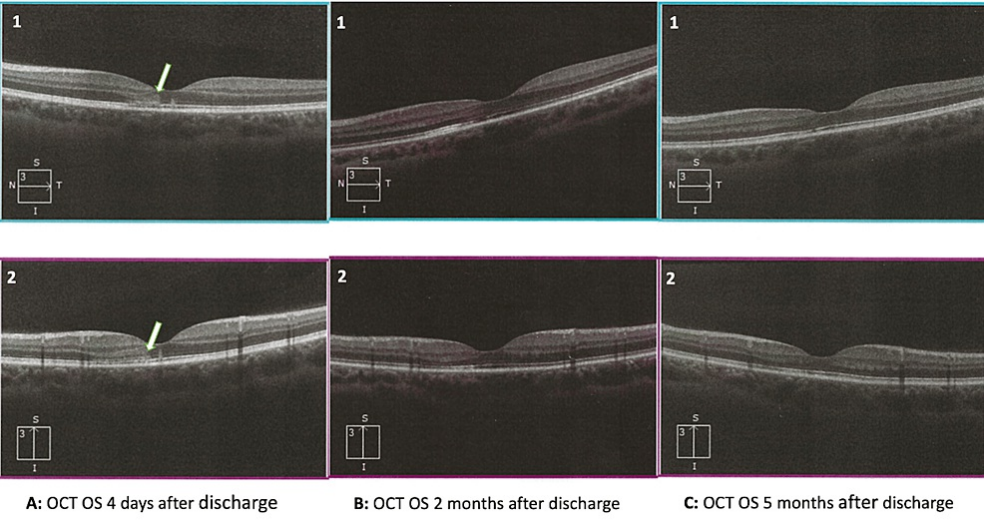
Clinical progression of acute macular neuroretinopathy as seen in cross-sectional optical coherence tomography (OCT). Cross-sectional optical coherence tomography (top images labeled 1 are horizontal sections while bottom labeled 2 are vertical sections of the affected left eye; N is nasal, T is temporal, S is superior, and I is inferior). (A) OCT of the left eye (OS) within a week of onset of vision changes; 1A & 2A white arrows point to focal hyperreflective bands at the level outer nuclear layer (ONL) and thickening of outer plexiform layer (OPL) with decreased reflectivity and disruption of the ellipsoid zone (EZ) near the nasal region as well as discontinuity of photoreceptors. (B & C) OCT OS approximately two months and four months, respectively, after the onset of symptoms showing resolution of the hyperreflective signal and near-complete recovery of the EZ attenuation.

**Figure 2:**
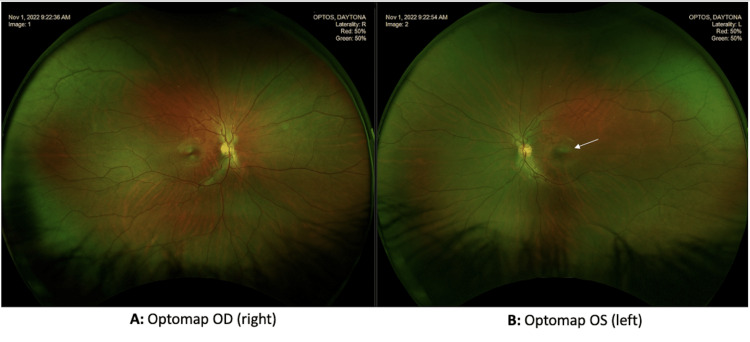
Optos® Optomap Ultra-widefield retinal fundus image taken roughly four months after the onset of vision changes. (A) Intact and well-demarcated macula, fovea, and the optic disc of the right eye. (B) A white arrow pointing to the ill-defined wedge-shaped macula of the left eye. Optos® Optomap Ultra-widefield (Dunfermline, UK).

**Figure 3:**
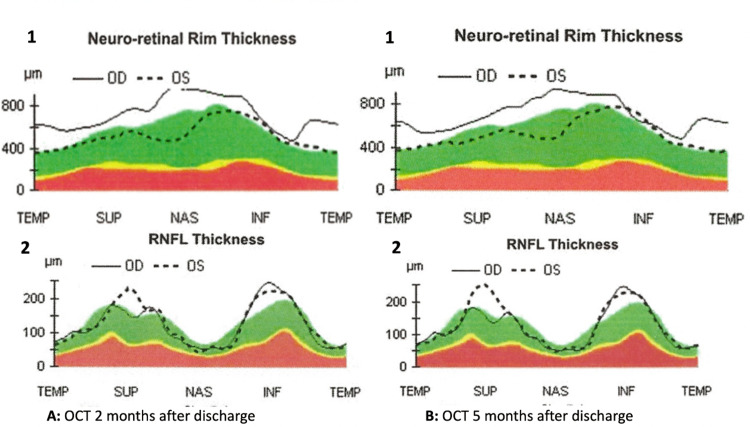
Zeiss OCT eye scan of the neuro-retinal rim thickness and retinal nerve fiber layer (RNFL). Quadrants temporal, inferior, and superior are displayed on the horizontal axis and the vertical axis thickness is measured in micrometers (μm). (1A & 2A) RNFL two months after the onset of symptoms. (1A & 2B) RNFL five months later. Both show normal thickness in both eyes in comparison to the average normative database noted in the green area; however, OS (dotted line) is slightly decreased in thickness. An intact double humph pattern is seen in 2A and 2B, which is seen in healthy eyes. These results indicate no damage to the ganglion cell axons of the optic nerve. OCT: optical coherence tomography; OS: left eye. Zeiss (Oberkochen, Germany).

The patient was started on aspirin 81 mg once a day with famotidine 40 mg once a day for stomach protection, which was discontinued one month after discharge. On the day of discharge, the patient started a seven-day steroid taper (prednisone 50 mg, 40 mg, 30 mg, 20 mg, 10 mg, 10 mg, and 5 mg). Diflucan 100 mg for three days was also given to start after steroid taper to prevent thrush. The patient was referred to a retina specialist for further evaluation with optical coherence tomography (OCT) to view the retina and optic nerve, which showed disorganization of the outer retina in the parafoveal region, consistent with her visual field defects and the wedge-shaped lesions seen on the exam, supporting a diagnosis of AMN in the left eye (Figure 1). Figure 2 is a retinal fundus image taken roughly four months after the onset of symptoms and a blurred macula in the left eye is shown. The patient was closely followed by a retina specialist with repeat OCT and retinal nerve fiber layer (RNFL) imaging (Figure 3) at two and five months after discharge, which showed no damage to the optic nerve, resolution of the hyperreflective signal in the macula, and near-complete recovery of the integrity of the outer nuclear layer and ellipsoid zone. No new treatments were added other than recommendations to stop or change birth control. Vision remained stable and improved to 20/25 OS and 20/20 OD. Per the outpatient gynecologist’s recommendation, the patient was switched to a mini-pill birth control medication, Incassia (norethindrone) 0.35 mg tablet once a day, roughly five months after discharge.

## Discussion

AMN is considered a rare ocular disease with an estimated prevalence of less than one in one million people; however, this is likely an underestimation as this disease is difficult to diagnose [7,8]. A literature review completed in 2016 found that there was a 35.6% association between oral contraceptive use and a 47.5% association between a nonspecific flu-like illness and AMN, in 101 identified cases [3]. AMN is also associated with other preceding risk factors like SARS-CoV-2 viral illness and vaccines developed to target the SARS-CoV-2 virus. In the literature, there has been an increased number of cases of AMN since the start of the SARS-CoV-2 pandemic [7,9-14]. It appears there are a number of AMN cases after SARS-CoV2 infection and the mRNA SARS-CoV-2 vaccines, with the hypothesized immune-mediated pathway mechanism triggering this macular disease [9,10]. In this case, the patient was vaccinated against SAR-CoV-2 roughly one year and three months prior to AMN symptoms, and had no side effects from the vaccine at that time, making this case less likely related to vaccine injury. Furthermore, the onset of visual symptoms of AMN began on day four of the SARS-CoV-2 infection suggesting that coronavirus was the cause.

SARS-CoV-2 is primarily known for causing severe respiratory syndrome; however, there is evidence that this virus is associated with multi-organ damage as the virus’s spike protein gains entry and function via the angiotensin-converting enzyme 2 (ACE-2) receptor, which is found throughout the body, in every central organ system [15]. ACE-2 receptors are also found in the venous and arterial endothelial cells in most organs, including the retina [16,17]. There is also evidence of retinal involvement as the SARS-CoV-2 viral particles have been found in the retina of autopsy samples [18]. The spike protein attaches to the ACE-2 receptor, which is found in the deep layers of the eye, including the retinal ganglion cell layer, inner plexiform layer, inner nuclear layer, and photoreceptor outer segments [19].

Our patient did have lymphocytopenia, a mildly elevated CRP, and elevated monocytes, all hallmark signs of an enhanced inflammatory cytokine storm. The pro-inflammatory mediators produced during a cytokine storm can cause a systemic activation of the coagulation pathways, which can lead to microthrombi, capillary obstruction, vascular leakage, cellular dysfunction, and subsequent tissue ischemia. The hypothesized mechanism of AMN includes a hyperinflammatory chain reaction leading to a microvascular thrombotic event and ischemia to the deep retinal capillary plexus. Thus, endothelial cell dysfunction and microvascular angiopathy after SARS-CoV-2 play a role in causing damage to the inner structures of the eye [20]. One study even found that SARS-CoV-2 can infect the retinal ganglion cells and photoreceptors by inducing the expression of several inflammatory genes, suggestive of an autoimmune process [6,21].

OCT is a non-invasive imaging tool that allows for precision diagnosis. It enables analysis and mapping of the retinal microvasculature by producing high-resolution cross-section images [22]. Characteristic signs of AMN were seen in our patient’s OCT, which showed hyperreflectivity in the outer nuclear level (ONL), outer plexiform layer (OPL), and disruption of the ellipsoid zone (EZ). The EZ is densely packed with mitochondria and therefore plays a critical role in photoreceptor health of the inner segment of the eye [23]. SARS-CoV-2 hijacks the host’s mitochondria, which functions in innate immune signaling, and dysfunction in the mitochondria leads to oxidative stress driving the production of proinflammatory cytokines [24]; this further illustrates that our patient’s systemic cytokine storm can also manifest at a cellular level. A prospective study done in Germany analyzed the microcirculation of the retina using OCT in patients and found macula micro-vessel vessel density changes in the plexiform layer, signifying a clinical marker for the severity of SARS-CoV-2 illness and indicating immune thrombosis. They further hypothesized that their findings correlate with systemic capillary microcirculation that can lead to the deterioration of organ function as a result of endothelial dysfunction, activation of the coagulation system, and overactivation of the immune system with cytokine release. They further suggest that immunosuppressing drugs like steroids might benefit prognosis [25]. Our patient did show improvement in vision after day two of high-dose IV steroids, signaling some benefit for treatment in patients that have AMN, but more research is needed to suggest steroids play a role in treatment for AMN. Our patient was also given remdesivir, which helped decrease SARS-CoV-2 viral replication and viral load; however, it is unclear if this antiviral played a role in the patient’s improvement as there are no previous reports showing remdesivir’s effectiveness in treating SARS-CoV-2 ocular manifestations.

Sex hormones are proven to affect the retina as there are estrogen and progesterone receptors located on the cornea, lens, retina, the central part of the retina known as the macula, and multiple other ocular tissues [26,27]. Furthermore, women on oral contraceptive pills (OCP) have a higher rate of cardiovascular events, ischemic strokes, and thromboembolic disease [28]. A study done in 2017 found thinner areas in the RNFL in those who were on a combination of estrogen and progesterone OCP [27]. One study found that the thickness of the retina and choroid has significant changes in women who were on OCP for more than a year [26]. It is important to note that the patient, in this case, was on the same brand of OCP for over six years with no previous issues. The timing of her AMN symptoms suggests that the SARS-CoV-2 infection was responsible for this incident and that her contraceptive pills were a risk factor that facilitated AMN. Currently, there are no treatment options for AMN, and the cessation of oral contraceptives is not known to affect the disease course of AMN. The patient was switched to a progestin-only pill (POP), or mini-pill, as these are known to have little effect on coagulation. She was also placed on aspirin for one month, which aided in some gradual improvement of her vision. Some reports suggest the initiation of anticoagulation may help revise the progression of ischemia-related SARS-CoV-2 damage, and short-term discontinuation of OCP should be considered to prevent further ischemic compromise [13]. This patient’s prognosis was good as the degree of reperfusion to the affected structures of the inner eye aided in correcting the vision back to a stable baseline of 20/25 in the left eye.

Overall, this case demonstrates that AMN is associated with SARS-CoV-2 infections. The proposed pathophysiology is a result of an inflammatory reaction leading to ischemia in the deep retinal capillary plexus. Furthermore, the spike protein has the capability to attach to the ACE-2 receptors of the retinal ganglion cell layer, inner plexiform layer, inner nuclear layer, and photoreceptors coupled with mitochondrial dysfunction. This case highlights the fact that SARS-CoV-2 is a vascular illness that can cause multi-organ injury, including ocular damage. Lastly, research studies would be helpful to confirm the association between AMN and SARS-CoV-2 and to help better understand treatment options for this etiopathogenesis of AMN. This study demonstrates the importance of prompt ophthalmology evaluation and the use of OCT to obtain an accurate diagnosis.

## Conclusions

This case of a 24-year-old female with no prior medical history, other than long-term oral contraceptive use, focuses on a patient who was diagnosed with a rare, acquired retinal disorder known as AMN. AMN in this patient was characterized by a left paracentral visual field deficit and a petaloid or wedge-shaped lesion noted in the macula. Antecedent to this event, the patient had an upper-respiratory-like illness and was found to be positive for SARS-CoV-2 infection. This case adds to the literature because it demonstrates the vascular pathophysiology behind SARS-CoV-2 and its ability to cause retinal vascular disease. Furthermore, it notes the importance of having ophthalmology conduct a thorough exam, including the use of multi-modal imaging such as OCT, as this can confirm the exact diagnosis. This patient only has minor visual changes and overall had resolution of her scotoma. AMN currently has no gold standard treatment, but understanding that this might be a vascular and/or immune-driven process might help with future research for treatment options that can aid in reperfusion.
